# Boosting Virology in Brazil

**DOI:** 10.1371/journal.pbio.0060057

**Published:** 2008-03-11

**Authors:** Maria Inês de Moura Campos Pardini, Leda Fátima Jamal, Edison Luiz Durigon, Eduardo Massad, José Fernando Perez, João Renato Rebello Pinho, Edward C Holmes, Paolo Marinho de Andrade Zanotto

## Abstract

The Viral Genetic Diversity Network, established in Brazil to enhance the study of viruses, antiviral drug resistance, and the spread of specific viral genotypes, also increased the net scientific production of virology research, and may provide a viable model for other developing countries to reduce the risks of pandemics and facilitate basic scientific research.

In 1997, in an effort to improve the pursuit of virology in the state of São Paulo, Brazil, a group of scientists proposed the establishment of a Viral Genetic Diversity Network (VGDN) to São Paulo's major scientific funding agency, Fundação de Amparo à Pesquisa do Estado de São Paulo (FAPESP). The major impetus for the project was the earlier establishment of the Xylella fastidiosa genome sequencing consortium [1], organised to counter this plant pathogen, which has had a serious impact on the Brazilian agricultural economy and industry. In addition to addressing this important national priority, the X. fastidiosa sequencing consortium also had a major qualitative impact on genome sciences in Brazil. Taking inspiration from the success of the genome sequencing effort, the VGDN envisaged a network of laboratories at several research institutions in the state of São Paulo, with the aim of studying key viral diseases within an applied context. Its main goal was to provide competence in genetic studies of viruses and to acquire primary data of immediate use (e.g., the monitoring of vaccines, antiviral drug resistance, and the spread of specific viral genotypes). The VGDN proposal was evaluated by a panel of top international scientists and eventually approved for funding by FAPESP.

Under the concept of a “virtual institute” fostered by FAPESP [2], the programme was organised as a multi-centre, multi-task network comprising five central laboratories and 17 satellite units, strategically distributed throughout the state. Although member institutions were encouraged to pursue their own independent lines of research, all had to cooperate on four viruses of particular public health importance for Brazil: HIV-1, hepatitis C virus, human respiratory syncytial virus (HRSV), and hantavirus. The unifying elements of the VGDN included: (1) a system of sampling and experimental design utilising clinical and epidemiological information; (2) large-scale automated viral gene sequencing; and (3) the tracking of genetic variants during epidemic outbreaks to detect variants of importance for diagnosis and vaccines.

A core set of VGDN laboratories started operations in 2001, conducting a pilot study on HIV-1 together with state-run sexually transmitted disease/AIDS reference centres at a total cost of US$2 million. Over the next few years, FAPESP invested a total of US$7.5 million in the VGDN. Crucially, the infrastructure, technical capabilities, and some research time provided under the VGDN were also utilised by each participating member as seed for the establishment of research projects beyond the four coordinated tasks. The VGDN had 41 researchers, the direct involvement of 115 graduate and 29 under-graduate students, and 69 technicians. Almost all of the institutions provided funds in addition to the resources included under the programme, and in three campuses across the state, new virology units (including new buildings) were established. A total of 20 laboratories (seven of which are biosafety level 3) were either newly built, renovated, or re-equipped.

The large amount of basic virological data resulting from each coordinated task has already been passed on to the public health system, and is being prepared for publication. The HIV-1 coordinated task has generated 20% of the viral genome, coupled with 495 pieces of socio-economic and clinical data from each of the 1,279 georeferenced patients from eight cities within the state of São Paulo sampled between 2001 and 2004. These data are currently being used for an intensive study of the patterns of dispersal of subtypes and circulating recombinants of HIV-1, as well as of resistance patterns relating to treatment outcome. As the Brazilian government spends US$731 million annually on free drug treatment and clinical follow-up for HIV-1 carriers, these unique data allow an in-depth assessment of the success of this important health programme, particularly in the face of considerable friction among pharmaceutical companies and the government on issues of drug prices and patents.

Similarly, the hepatitis C virus coordinated task involves the study of 1,000 patients undergoing antiviral therapy in three cities. The data produced as part of this project have provided vital information on the role of blood donation policies in the dispersal of viral genotypes over the last 20 years. Since 2005, a total of 2,000 samples have been collected for the HRSV coordinated task, which, combined with retrospectively sampled sequence data, has provided a unique insight into the intricate fluctuating dynamics of HRSV-type genotypes A and B. Finally, hantavirus pulmonary syndrome is an increasing health problem in Brazil (884 cases were reported in 2007, with a 39% case fatality rate [3]), due to the encroachment of urban and agricultural land use on the habitats of the rodents who serve as reservoirs for the virus. With the data collected under the hantavirus coordinated task, researchers are studying the association of human cases with specific viral lineages.

In addition to all of these direct benefits accrued from the operation of the VGDN, the indirect impact on the net scientific output in the state of São Paulo has been extensive. This is evident from PubMed searches for publications by virologists based in São Paulo state. A remarkable change in the annual rate of publishing was observed from 2001 onwards (Figure 1). As a comparison, we also plotted the equivalent data from the neighbouring state of Rio de Janeiro, which harbours the largest community of virologists in Brazil. The slopes of the lines on the graph up to 2001 are indistinguishable. However, after 2001, the publication rate in São Paulo showed a considerable increase, which can be attributed to an overall boost in virological expertise due to the VGDN. A similar growth was also observed for biomedical sciences in São Paulo, although no VGDN groups directly invested resources in these efforts.

**Figure 1:**
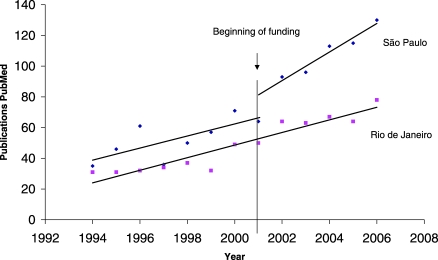
The Number of Virology Publications, Documented on PubMed, from 1992–2006 for the States of São Paulo and Rio de Janeiro, Brazil A major increase in publication numbers was observed in São Paulo following the onset of VGDN funding in 2001.

It is clear that the VGDN has been pivotal in establishing a more efficient infrastructure for the surveillance and monitoring of emerging and reemerging viral diseases in São Paulo state, using modern techniques and facilities and bringing public health experts and academic researchers together under the same virtual roof. We believe that this unique initiative may constitute a viable model for other developing countries, not only to help alleviate the risk of pandemics, but also to facilitate and support basic scientific research.

## Abbreviations

FAPESP: Fundação de Amparo à Pesquisa do Estado de São Paulo
HRSV: human respiratory syncytial virus
VGDN: Viral Genetic Diversity Network
